# Entomopathogenic Fungi-Mediated AgNPs: Synthesis and Insecticidal Effect against *Plutella xylostella* (Lepidoptera: *Plutellidae*)

**DOI:** 10.3390/ma15217596

**Published:** 2022-10-28

**Authors:** Tárcio S. Santos, Camila de Souza Varize, Elena Sanchez-Lopez, Sona A. Jain, Eliana B. Souto, Patrícia Severino, Marcelo da Costa Mendonça

**Affiliations:** 1 Post-graduate Program in Industrial Biotechnology (PBI), University Tiradentes (Unit), Av. Murilo Dantas, 300, Aracaju 49010-390, Brazil; 2 Sergipe Agricultural Development Company (Emdagro), Av. Carlos Rodrigues da Cruz s/n, Aracaju 49081-015, Brazil; 3 Department of Pharmacy, Pharmaceutical Technology and Physical Chemistry, Faculty of Pharmacy and Food Sciences, University of Barcelona, 08028 Barcelona, Spain; 4 Institute of Nanoscience and Nanotechnology (IN2UB), University of Barcelona, 08028 Barcelona, Spain; 5 Unit of Synthesis and Biomedical Applications of Peptides, IQAC-CSIC, 08034 Barcelona, Spain; 6 Institute of Technology and Research (ITP), Av. Murilo Dantas, 300, Aracaju 49010-390, Brazil; 7 Department of Pharmaceutical Technology, Faculty of Pharmacy, University of Porto, Rua de Jorge Viterbo Ferreira, 228, 4050-313 Porto, Portugal; 8 REQUIMTE/UCIBIO, Faculty of Pharmacy, University of Porto, Rua de Jorge Viterbo Ferreira, 228, 4050-313 Porto, Portugal

**Keywords:** biological synthesis, silver nanoparticles, entomopathogenic fungi, agricultural pest control, biological control

## Abstract

The insect *Plutella xylostella* is known worldwide to cause severe damage to brassica plantations because of its resistance against several groups of chemicals and pesticides. Efforts have been conducted to overcome the barrier of *P. xylostella* genetic resistance. Because of their easy production and effective insecticidal activity against different insect orders, silver nanoparticles are proposed as an alternative for agricultural pest control. The use of entomopathogenic fungi for nanoparticle production may offer additional advantages since fungal biomolecules may synergistically improve the nanoparticle’s effectiveness. The present study aimed to synthesize silver nanoparticles using aqueous extracts of *Beauveria bassiana*, *Metarhizium anisopliae,* and *Isaria fumosorosea* isolates and to evaluate their insecticidal activity against *P. xylostella*, as innovative nano-ecofriendly pest control. The produced silver nanoparticles were evaluated by measuring the UV–vis spectrum and the mean particle size by dynamic light scattering (DLS). *I. fumosorosea* aqueous extract with 3-mM silver nitrate solution showed the most promising results (86-nm mean diameter and 0.37 of polydispersity). Scanning electron microscopy showed spherical nanoparticles and Fourier-Transform Infrared Spectroscopy revealed the presence of amine and amide groups, possibly responsible for nanoparticles’ reduction and stabilization. The CL_50_ value of 0.691 mg mL^−1^ was determined at 72-h for the second-instar larvae of the *P. xylostella*, promoting a 78% of cumulative mortality rate after the entire larval stage. From our results, the synthesis of silver nanoparticles mediated by entomopathogenic fungi was successful in obtaining an efficient product for insect pest control. The *I. fumosorosea* was the most suitable isolate for the synthesis of silver nanoparticles contributing to the development of a green nanoproduct and the potential control of *P. xylostella*.

## 1. Introduction

The diamondback moth *Plutella xylostella* (Lepidoptera: *Plutellidae*) is a microlepidopteran, popularly known as the cabbage moth. This species is one of the most studied insect pests in the world and is reported to cause extensive damage to vegetable crops of the *Brassicaceae* family, including cabbage, cauliflower, mustard, and rapeseed, among others [1]. The damage caused by this insect to the host plant is related to the feeding habits in the larval stage. The caterpillars tend to scrape the parenchyma tissue, making holes in the leaf surface and reducing the photosynthetically active area. Besides this, its high rate of reproduction (e.g., more than 20 generations per year in tropical regions) increases plant damage quite rapidly, making the entire crop harvesting unfeasible, if no containment measures are adopted. In addition, the low incidence of natural enemies and the high genetic variability of this insect facilitate/promotes the development of chemical pesticide resistance [2,3].

The control of *P. xylostella* costs a minimum of USD 1.4 billion per year globally, reaching USD 5 billion when considering crop productivity loss [2]. Therefore, the main challenge is to overcome the high resistance against chemical pesticides since this moth is one of the most resistant and destructive pests worldwide. Due to the high rate of reproduction, the intense and fast infestation caused by this plague increases the need to overcome these obstacles [4]. Nanotechnology is providing significant benefits to the agrifood sector, including the delivery of nutraceuticals and nanopesticides, increasing productivity, and favoring plant management and development, to ultimately increase food safety [5]. Among available alternatives, silver nanoparticles emerge as a potential product for use in insect pest control, since they are easy to synthesize and handle, and also present insecticidal/antimicrobial actions [6,7,8,9]. However, the main concern involving the production of nanomaterials is the generation of toxic waste. Thus, the development of these products requires more sustainable, green ways to reach higher profits and safer synthesis. The synthesis of silver nanoparticles by biological process is attracting increasingly interest because it represents a simple, sustainable, and low-cost method [6,7].

Silver nanoparticles synthesized by a biological process have toxicity against insects, being the application mainly directed against mosquitoes [10]. The use of algae extracts to produce silver nanoparticles was already reported to control the insect *P. xylostella* [11], however, there is no evidence for the effectiveness of silver nanoparticle synthesis mediated by entomopathogenic fungi for the same purpose.

Therefore, this work aims to synthesize silver nanoparticles with an aqueous extract of entomopathogenic fungi and to evaluate their insecticidal action against *Plutella xylostella*. We report the synthesis of silver nanoparticles using different species of entomopathogenic fungi. The influence of the silver nitrate concentration on nanoparticle formation is also presented. The nanoparticles were characterized by UV-Vis spectroscopy, Dynamic Light Scattering (DLS), Fourier Transform Infrared Spectroscopy (FTIR), and Scanning Electron Microscopy (SEM). Finally, from the bioassays using the nanomaterial selected, we showed the sublethal concentrations and the effect of the LC_50_ in the life-cycle stages of *P. xylostella*.

## 2. Materials and Methods

**Figure 1:**
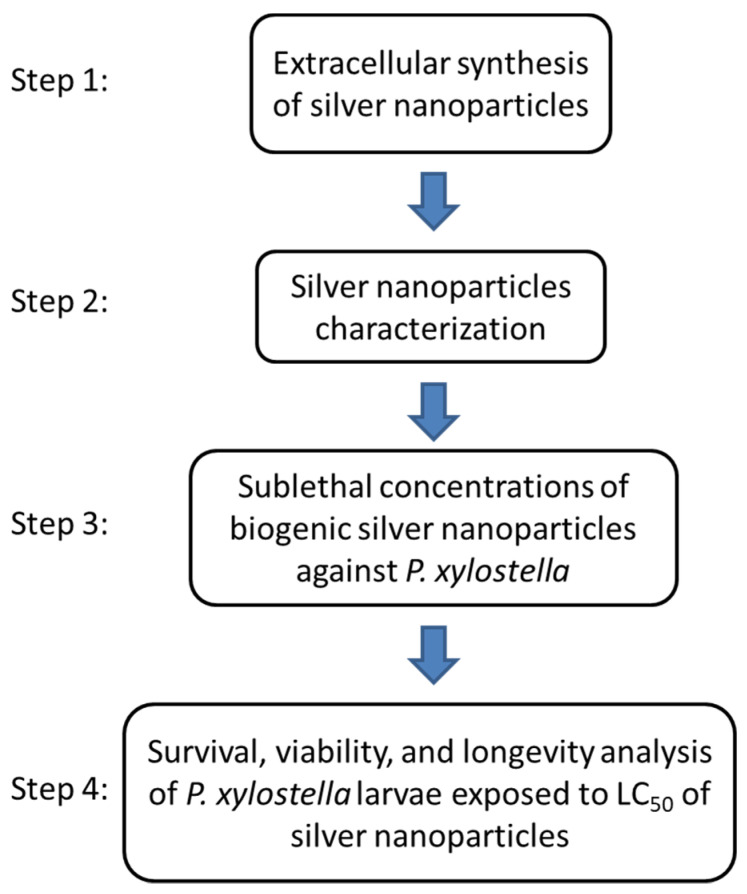
Experimental procedure flowchart.

The experimental procedure followed the sequence described in Figure 1.

### 2.1. Entomopathogenic Fungal Isolates

The four fungal isolates used to produce silver nanoparticles belong to the species *Beauveria bassiana* (Bals.—Criv.) Vuill. (*Hypocreales: Cordycipitaceae*) strains SE109 and 1260, *Metarhizium anisopliae* (Metschn.) Sorokīn (*Hypocreales: Clavicipitaceae*) SE202, and *Isaria fumosorosea* Wize (*Hypocreales: Cordycipitaceae*) SE301 (Table 1). The fungi are kept in colonized potato dextrose agar (PDA) disks (5-mm-diameter), and stored in cryogenic tubes containing 10% of a glycerol solution, at −20 °C.

### 2.2. Extracellular Synthesis of Silver Nanoparticles

The isolates were grown on a PDA medium at a temperature of 25 ± 2 °C, for 7 days. For their use, colonized PDA disks (5-mm-diameter) were inoculated into potato dextrose broth (PDB) and incubated for 7 days at 25 ± 2 °C on an orbital shaker (100 rpm). Next, the fungal biomass was filtered through filter paper (Whatman No. 1, London) and washed three times with ultrapure water. Then 10 g of the washed biomass was added to an Erlenmeyer flask containing 100 mL of ultrapure water, maintained for 72 h at 25 ± 2 °C on an orbital shaker (100 rpm). Then, 90 mL of the liquid extracted (aqueous extract) from a new filtering was mixed with 10 mL of 1, 3, and 5 mM of AgNO_3_ solution. This mixture was incubated in the dark for 96 h at 25 ± 2 °C on an orbital shaker (100 rpm) [6,12]. 

### 2.3. Silver Nanoparticles Characterization

The formation of silver nanoparticles was monitored by UV-Visible spectroscopy (DR 5000 spectrophotometer, Hexis Científica, Jundiaí, São Paulo, Brazil,) in the spectral region between 300 and 800 nm. The absorbance values were plotted in graphs using the Origin Pro 2019b software (OriginLab Coorporation, Northampton, MA, USA, 2019). The nanoparticle diameter and Polydispersity Index (PDI) were determined by Dynamic Light Scattering (DLS) analysis using the Zetasizer Nano Instrument (Malvern, Australia, model Nano-S). The selected nanoparticles were characterized by Scanning Electron Microscopy (SEM) (JEOL microscope, model JSM-IT200, Tokyo, Japan). The functional groups present in the silver nanoparticles were analyzed by Attenuated Total Reflection-Fourier Transform Infrared (ATR-FTIR) spectroscopy (Agilent Technologies, Agilent Cary 630, Santa Clara, CA, USA), equipped with a ZnSe-diamond composite crystal accessory. The spectra were collected over a wavenumber range from 4000–600 cm^−1^ with a spectral resolution of <2 cm^−1^. Agilent MicroLab PC and Origin Pro 2019b software were used for data gathering and transmittance graph plotting, respectively (OriginLab, 2019).

### 2.4. Sublethal Concentrations of Biogenic Silver Nanoparticles against P. xylostella

Cabbage leaf discs (*Brassica oleracea* L.) (8-cm-diameter) were submerged in the suspension of silver nanoparticles (previously dispersed in an ultrasonic bath for 10 min) at concentrations of 0.1; 0.3; 0.7; 0.9 and 1.2 mg mL^−1^ and maintained at room temperature (26 ± 2 °C) till complete drying. Then, one cabbage leaf disk was transferred to a Petri dish (90 × 10 mm), which received 25 second-instar larvae of *P. xylostella* (emerged at 72 h). The Petri dishes were sealed and maintained in an acclimatized room (T = 26 ± 2 °C, RH = 60 ± 10%, and photophase = 12 h) for a 72-h period to assess the mortality rate. The commercial pesticide Deltamethrin (Decis 25 EC) was also evaluated at concentrations of 0.0075, 0.0225, 0.0525, 0.5, and 0.75 mg mL^−1^, applying the same method as mentioned above. Five replicates were tested for the silver nanoparticles and Deltamethrin treatments. Since Tween^®^ 80 0.05% was used to homogenize nanoparticle suspension used in the bioassay prior to the application of silver nanoparticles in the insect, the same solution of Tween^®^ 80 0.05% was used as a negative control. 

### 2.5. Survival, Viability, and Longevity Analysis of P. xylostella Larvae Exposed to LC_50_ of Silver Nanoparticles

Cabbage leaf discs (8-cm-diameter) were submerged into a suspension of silver nanoparticles corresponding to the CL_50_, following the same method/conditions of the sublethal concentrations test above described. Each Petri dish (90 × 10 mm) received one cabbage leaf disk and 25 second-instar larvae of *P. xylostella* (emerged at 72 h), also maintained in an acclimatized room (T = 26 ± 2 °C, RH = 60 ± 10% and photophase = 12 h). The larvae were transferred to a new Petri dish containing one untreated cabbage leaf disc every 72 h, during the entire evaluation. The caterpillar mortality was evaluated daily until it reaches 100% or enter the pupal stage. The percentage of viable insects and the time taken in each phase of the biological cycle were also determined, until the pupal stage. In total, 10 replicates were tested for this bioassay and a solution of Tween 80^®^ 0.05% was used as a negative control.

### 2.6. Statistical Analysis

The cumulative mortality of the insects after the third day of exposure to the treatment was submitted to the Probit analysis to estimate the lethal concentration (LC). The daily mortality values were used to estimate the lethal time (LT) and to construct a survival curve by the Kaplan–Meier method (Log-rank—Mantel-Cox). The number of insects that passed to the pupal phase as well as the duration time of each biological cycle phase was quantified. The parameters of larval viability, larval duration, pupal viability, and pupal duration after exposure to LC_50_ of silver nanoparticles were analyzed. The results were submitted to the t-test. The statistical analyzes were performed using SPSS software version 23 (IBM Corp., Armonk, NY, USA, 2015). The graphs of LC were made by the GraphPad Prism 8.0.1. software (GraphPad 8.0.1. Software, 2018).

## 3. Results and Discussion

### 3.1. Synthesis of Silver Nanoparticles 

**Figure 2:**
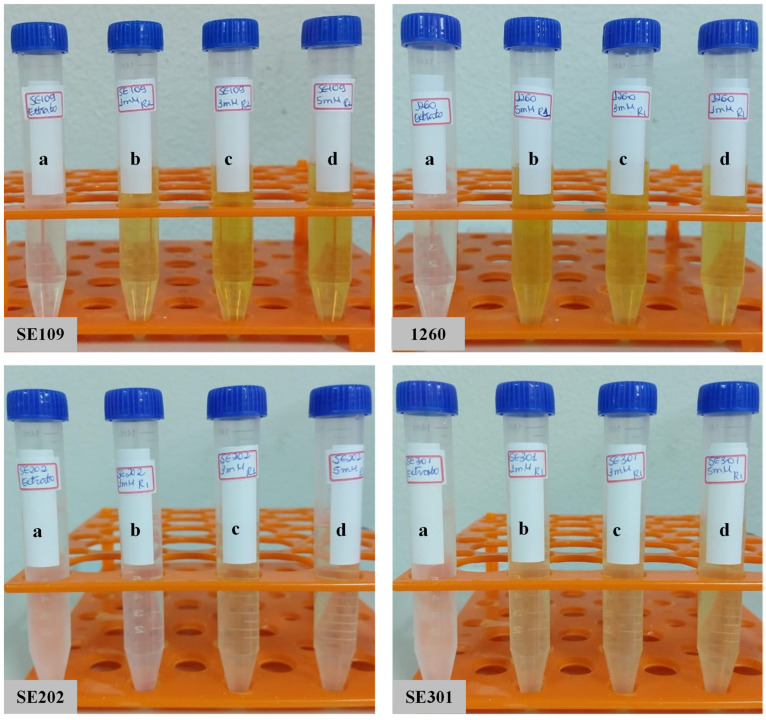
Synthesis reaction of silver nanoparticles with aqueous extract of entomopathogenic fungi isolates and variation in silver nitrate concentration. SE109 e 1260 (*B. bassiana*), SE202 (*M. anisopliae*) and SE301 (*I. fumosorosea*). (a) Pure extract, (b) AgNO_3_ 1 mM, (c) AgNO_3_ 3 mM, (d) AgNO_3_ 5 mM.

**Figure 3:**
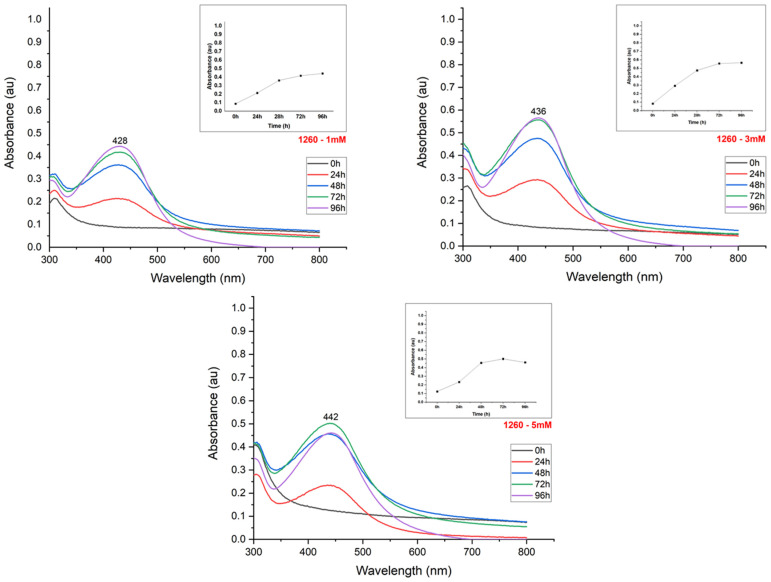
UV-Visible absorption spectrum of the evaluation of the effect of silver nitrate concentration on the synthesis of silver nanoparticles using an extract of the fungus *B. bassiana* (isolate 1260).

**Figure 4:**
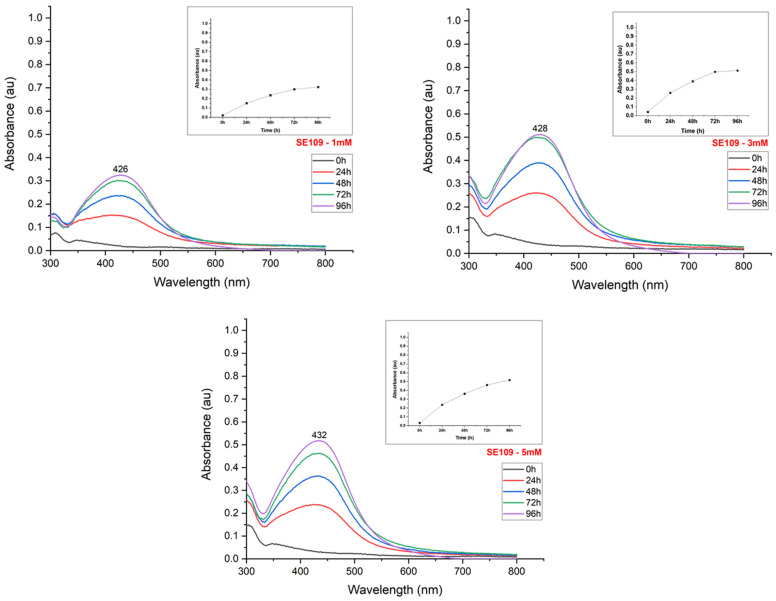
UV-Visible absorption spectrum of the evaluation of the effect of silver nitrate concentration on the synthesis of silver nanoparticles using an extract of the fungus *B. bassiana* (isolate SE109).

**Figure 5:**
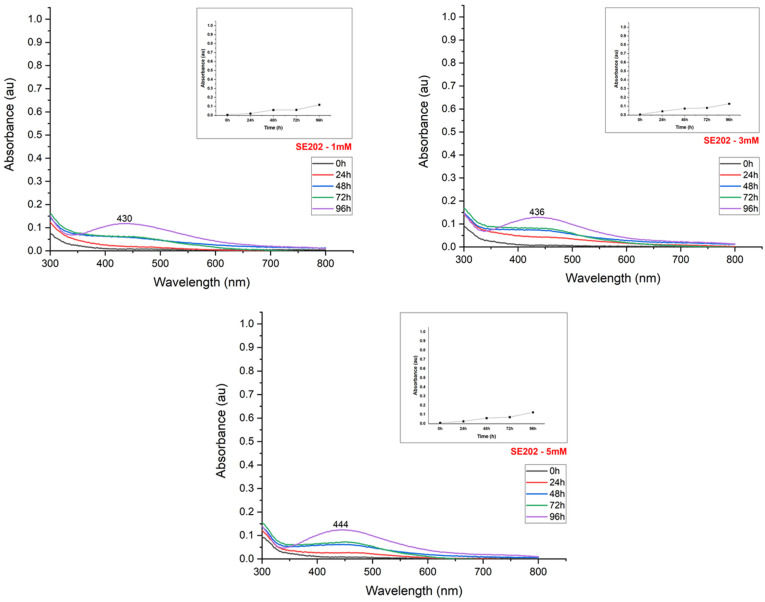
UV-Visible absorption spectrum of the evaluation of the effect of silver nitrate concentration on the synthesis of silver nanoparticles using an extract of the fungus *M. anisopliae* (isolate SE202).

**Figure 6:**
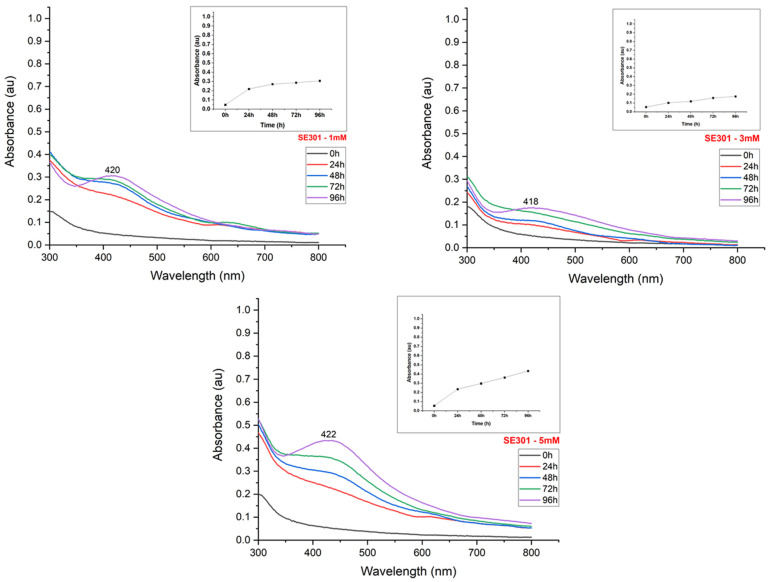
UV-Visible absorption spectrum of the evaluation of the effect of silver nitrate concentration on the synthesis of silver nanoparticles using an extract of the fungus *I. fumosorosea* (isolate SE301).

The synthesis reaction caused a color change from light to dark yellow in the suspensions, according to the silver nitrate content used for nanoparticle production (Figure 2). The UV-Visible spectroscopy exhibited peaks between 400–500 nm, confirming the reduction of the silver salt and the effective formation of nanoparticles (Figure 3, Figure 4, Figure 5 and Figure 6). The absorption peaks increased in concomitance with the higher concentrations of AgNO_3_, observed in 1260, SE109, and SE301 aqueous extract (Figure 3, Figure 4 and Figure 6), being this relationship not observed in SE202 (Figure 4). Previously, it has been reported that the silver ions content affects the reaction time of the nanoparticle synthesis. A previous study used the *Aspergillus oryzae* extract in the synthesis of the silver nanoparticles and reported that although the speed of the reaction increases at higher concentrations of AgNO_3_, the size of particles can also increase (9 and 10 mM) [13]. The optimization of the synthesis rate was achieved when the silver nitrate content was increased from 1 mM to 1.5 mM, using *Aspergillus fumigatus* extract in the nanoparticle synthesis reaction [14]. Therefore, it is suggested that the higher availability of silver ions affords the improvement of the nanoparticle formation in different aspects, such as the reaction time and synthesis rate. On the other hand, the excess of silver ions induces a delay in nanoparticle formation and affects the particle size. The silver nitrate above 8 mM was reported to cause a reduction in nanoparticle formation, using the fungus *Talaromyces purpurogenus* (*Eurotiales: Trichocomaceae*) extract [15]. 

The reduction in the nanoparticle synthesis was also observed at 96 h in the presence of 5 mM silver nitrate using isolate 1260 (Figure 3). The broad peak in the absorption spectrum and the decrease in absorbance indicate the formation of agglomerates, aggregates, or even nanoparticle dissolution. The imbalance between the concentration of the reducing agent and silver ions suggests the denaturation of biomolecules or the inefficiency of the reducing agents to form new nanoparticles [16]. 

The maximum absorbance evaluation (UV-Visible absorption spectrum) allowed us to estimate the nanoparticle synthesis rate according to the incubation time (Figure 3, Figure 4, Figure 5 and Figure 6). The nanoparticle formation stabilized at 96 h in the presence of AgNO_3_ 3 mM and 1260, SE109, and SE301 extracts, allowing sufficient time for the silver ions depletion. The most suitable incubation time was observed at 72 h in the presence of AgNO_3_ 5 mM and 1260 aqueous extract for the nanoparticle synthesis. The nanoparticle synthesis rate depends mainly on the characteristics of the metabolite responsible for the reduction of silver. A study reported the absorbance stabilization peak at 8 min of incubation using AgNO_3_ 1 mM in the presence of *Ocimum sanctum* extract in the nanoparticle synthesis [17]. Another study showed an increase in nanoparticle formation at 48 h using the extract of cyanobacteria *Oscillatoria limnetica* [18]. Differences in the incubation time are expected for the biological synthesis of silver nanoparticles once extracts from different organisms present quantitative and qualitative differences in their compounds.

### 3.2. Silver Nanoparticle Characterization

The silver nanoparticles formed in the aqueous extracts of 1260 (*B. bassiana*) and SE202 (*M. anisopliae*) showed larger diameters, according to the increase of AgNO_3_. The opposite was observed in the SE109 (*B. bassiana*), presenting a smaller diameter of nanoparticles in a higher concentration of AgNO_3_. The AgNO_3_ 3 mM in the presence of the SE301 (*I. fumosorosea*) aqueous extract formed the smallest size nanoparticles of 86.26 nm diameter (Table 2). 

The polydispersity index was not affected by the different concentrations of AgNO_3_. The silver nanoparticles synthesized with 1 mM + SE202 and 3 mM + SE301 showed the lowest polydispersity index, with values of 0.27 and 0.37, respectively. (Table 2). Therefore, these combinations (1 mM + SE202 and 3 mM + SE301) produced the most homogeneous particles size. The other reactions produced nanoparticles with a polydispersity index ranging from 0.41 to 054, presenting medium values of polydispersity [19].

The production and stabilization of silver nanoparticles occur through the interaction between metabolites present in the biological extract and silver ions. The extract concentration and the type of molecule involved in the nanoparticle synthesis (protein, pigment, toxin, among others) affect the number of functional groups available to promote the metal ions reduction [13,20]. However, there is no consensus on which biomolecule is responsible for the synthesis process, and there may be different enzymes involved in the biosynthesis of metallic nanoparticles depending on the species or fungal isolate used. Studies on the mechanism of formation of silver nanoparticles by fungal metabolites report the influence of functional groups of proteins and amino acid residues in the synthesis of silver nanoparticles by *B. bassiana* and *I. fumosorosea* [21,22], oxidoreductases enzymes during the synthesis of these materials by *Metarhizium robertsii* [23] and the action of nitrate reductase enzyme and NADPH-dependent enzymes and quinones in the mediated synthesis by *Fusarium oxysporum* [24]. Thus, the imbalance between the functional groups and metal ions reduces the reaction efficiency, forming larger particles, as observed in the 1260 and SE202 extracts. The SE109 extract showed particle size reduction even in the higher concentrations of AgNO_3_, indicating the presence of more efficient metabolites for nanoparticle formation. The presence of 3 mM AgNO_3_ in the SE301 extract promoted the smallest nanoparticles formation and also more homogeneous particle size, it may be related to the better distribution between the reducing agent and silver ions in the reaction, once these aspects were not observed in other concentrations of AgNO_3_. Therefore, the nanoparticles synthesized in the presence of AgNO_3_ 3 mM + entomopathogenic fungi *I. fumosorosea* (SE301) aqueous extract were used in the next stages of the work, for their characterization (FTIR and SEM) and toxicity evaluation against *P. xylostella*.

**Figure 7:**
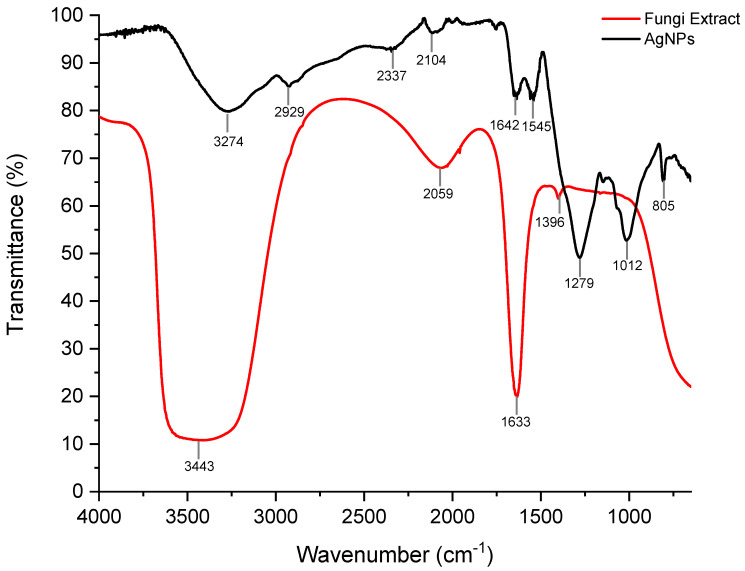
Fourier Transform Infrared Vibrational Spectroscopy (FTIR) spectrum showing band patterns of the aqueous extract of the fungus *I. fumosorosea* (red) and of AgNPs synthesized with the fungal extract (black).

The FTIR analysis of the silver nanoparticles (formed in the combined presence of *I. fumosorosea* aqueous extract + AgNO_3_ 3mM) showed characteristic bands in the spectral regions of 3400–2400, 2260–2100, 1680–1630, 1450–1375, 1350–1000 and 805 cm^−1^ (Figure 7), indicating the presence of N-H, C-N, C=O, C-H, and C≡C functional groups, which may be related to the presence of proteins or amino acid residues present in the fungal extract—being these molecules possibly responsible for the reduction of silver ions and the formation of nanoparticles [22,25,26].

The band shift of the fungal extract spectrum at 3443 and 1633 cm^−1^ regions compared to the silver nanoparticles spectrum can be attributed to the bond breaking of amine and amide groups, which are associated with the interaction in the silver nanoparticle formation. The interaction between amine/amide groups and silver nanoparticles in the biological route was previously reported, using plants and fungal extracts [10,27,28,29]. In this context, we suggest that the amine and amide groups present in the *I. fumosorosea* extract may be the precursors of the silver ions reduction, supporting the synthesis of the silver nanoparticles. 

**Figure 8:**
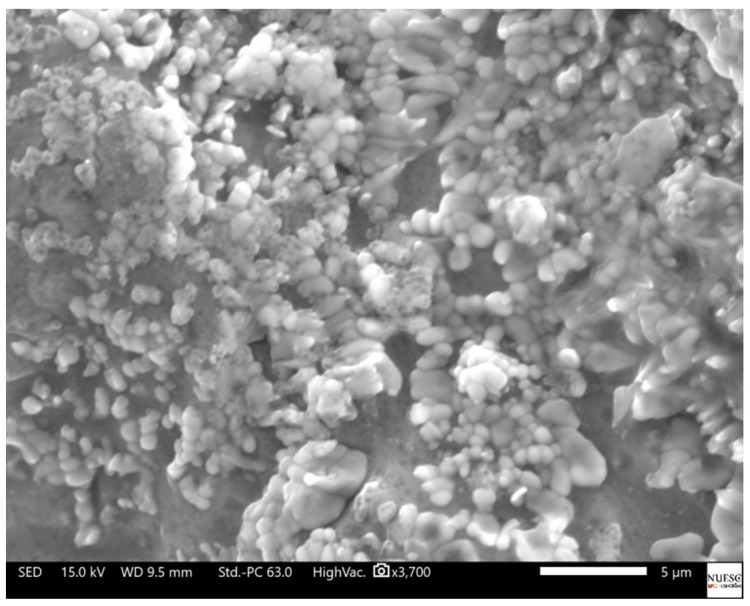
Scanning electron microscopy of silver nanoparticles synthesized with *I. fumosorosea* extract and 3 mM silver nitrate.

The SEM analysis of the silver nanoparticles allowed the visualization of circular morphology predominance, showing varied particle sizes adhered to the matrix of the lyophilized fungal extract (Figure 8). The morphology and diameter variation of the silver nanoparticle is influenced by the extract concentration of the microorganism used in the reaction (i.e., reducing agent), as well as the proportion of silver ions available in the reaction [29,30]. 

### 3.3. The Lethal Concentration of Biogenic Silver Nanoparticles against P. xylostella

**Figure 9:**
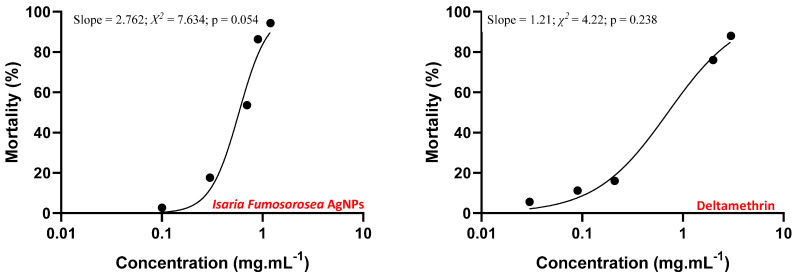
Probit curve of *Plutella xylostella* exposed to silver nanoparticles synthesized with the entomopathogenic fungus *Isaria fumosorosea* (**left**) and Deltamethrin (**right**). Residual effect after 72 h.

Silver nanoparticles at different concentrations were toxic against *P. xylostella*, showing a significant variation in the results (F_4.20_ = 53.967; *p* = 0.000). Thus, it was possible to estimate the sublethal concentrations using a probit procedure. (Figure 9). The LCs estimated values used for the silver nanoparticles action against *P. xylostella* were LC_30_ = 0.144 mg mL^−1^, LC_50_ = 0.691 mg mL^−1^, and LC_90_ = 2.011 mg mL^−1^, considering an angular coefficient (line slope) of 2.762 (Table 3).

Some studies reported the lethal concentration of the silver nanoparticles for *P. xylostella*. The silver nanoparticles synthesized using a marine macro red algae *Hypnea muciformis* showed high toxicity on *P. xylostella* in the larval and pupal stages, presenting LC_50_ = 26.47 mg L^−1^ for second instar larvae at 96 h of exposure [11]. The silver nanoparticles produced from ethanolic extracts of ginger (*Zingiber officinale*), Indian neem (*Azadirachta indica*), fig tree (*Datura stramonium*), bitter melon (*Momordica charantia*), clove (*Syzygium aromaticum*), cinnamon (*Melia azedarach*), eucalyptus (*Eucalyptus camaldulensis*) and garlic (*Allium sativum*) also presented toxic effects on *P. xylostella* third instar larvae, showing LC_50_ ranging from 0.337 mg mL^−1^ (fig tree) to 0.729 mg mL^−1^ (garlic) at 72 h of exposure [31]. The LCs values of silver nanoparticles synthesized using *I. fumosorosea* extract have not yet been reported on for the different life cycle stages of *P. xylostella*. The silver nanoparticle LCs variation previously reported may occur due to the different nanoparticle physicochemical characteristics and also because of the molecules involved in the nanoparticle synthesis and capping. The molecules from extracts or even adhered to the biogenic nanoparticle can directly influence the biological action, affecting the toxicity [32,33]. The LC_50_ value (0.691 mg mL^−1^) of the silver nanoparticles was higher compared to Roni et al. (2015) (26.47 mg L^−1^) [11], and within the range compared to Ali et al., (2019) [31] (0.337–0.729 mg mL^−1^). 

The *P. xylostella* populations have high genetic variability and toxicity resistance to several chemical compounds [34]. The toxic effect of the commercial insecticide Deltamethrin (Decis 25 EC) on *P. xylostella* was estimated in order to compare the LCs results of the silver nanoparticles toxicity tests. The different concentrations of the Deltamethrin showed significant difference in the mortality of *P. xylostella* (F_4.20_ = 56.864; *p* = 0.000), presenting LC_30_ = 0.009 mg mL^−1^; LC_50_ = 0.301 mg mL^−1^, and LC_90_ = 3.427 mg mL^−1^, considering an angular coefficient (line slope) of 1.214 (Figure 9; Table 3). Deltamethrin is an insecticide belonging to the pyrethroid family, indicated in the control of *P. xylostella* at a concentration of 0.0075 mg mL^−1^. The CL_30_ value (0.009 mg mL^−^1) estimated in our study is approximate to the concentration indicated for Deltamethrin application. Other study presented the values of LC_50_ = 0.332 mg mL^−1^ (0.321–0.345) and LC_90_ = 0.436 mg mL^−1^ (0.411–0.473) to control *P. xylostella* using Deltamethrin [35], that is, approximate value of LC_50_ presented in our study, but different comparing to the CL_90_. The present study presented a slower action of Deltamethrin LC_90_ on *P. xylostella*, possibly due to adaptation and better resistance to this chemical. The difference in the toxicity results from the application of silver nanoparticles (from AgNO_3_ 3 mM + entomopathogenic fungi *I. fumosorosea*) and Deltamethrin may occur because of their different modes of action. Deltamethrin acts against insects via ingestion and direct contact, promoting neurotoxicity through the interaction with sodium channels, present in the neurological cells. The frequent use of Deltamethrin promotes mutations in insect structures, contributing to its resistance [36,37]. Silver nanoparticles can also act against insects via ingestion and direct contact, presenting toxicity through different routes, promoting cuticle damage, and oxidative stress and reducing enzymatic activity (acetylcholinesterase and Cu-dependent enzymes). These different routes of toxicity can act simultaneously, favoring the action of the silver nanoparticles against the insect and reducing the possibility of insect resistance. Therefore, the use of silver nanoparticles is an advantageous alternative in the control of insect pests, such as *P. xylostella*, which is resistant to several chemical pesticides [38,39,40,41,42,43]. In addition, Deltamethrin is highly toxic to humans and other animals and may cause the development of cellular anomalies. In contrast, silver nanoparticles can be synthesized by environmentally sustainable techniques, such as synthesis using fungal extracts, which improve their biocompatibility [44,45]. However, the interaction of AgNPs with plant tissues, their post-harvest preservation, and also their interaction with the human gastrointestinal tract, must be considered to ensure the safe use of such materials [46,47].

### 3.4. Survival, Viability, and Longevity Analysis of P. xylostella Larvae Exposed to LC_50_ of Silver Nanoparticles

**Figure 10:**
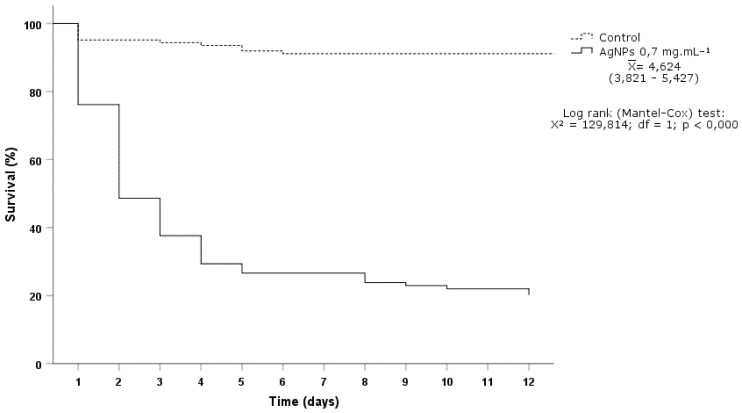
Survival curve of *Plutella xylostella* caterpillars treated with *Isaria fumosorosea* AgNPs.

The survival of *P. xylostella* caterpillars treated with silver nanoparticles (from AgNO_3_ 3 mM + entomopathogenic fungi *I. fumosorosea*) at LC_50_ (0.691 mg mL^−1^) showed different results compared to the control group (Log-rank Mantel-Cox: X^2^ = 129.814; *p* = 0.000). The insects treated with silver nanoparticles presented a median lethal time (LT_50_) of 4.624 days (3.821–5.427) and 78.25 ± 11.04% (F_1.8_ = 192.627; *p* = 0.000) of cumulative mortality over the entire larval period (Figure 10).

The silver nanoparticles (from AgNO_3_ 3 mM + entomopathogenic fungi *I. fumosorosea*) showed effective toxicity against *P. xylostella* caterpillars. Significant insecticidal action was previously reported by the exposure of biogenic silver nanoparticles on *P. xylostella* (96 h) [11]. The time required for the establishment of toxicity is variable, as it depends on the nanomaterial characteristics and insect physiology [40].

The viability of *P. xylostella* caterpillars treated with silver nanoparticles showed a significative difference (T = 13.879; *p* = 0.000), However, significative differences were not observed in the duration of larval and pupal stages (days) and in the pupal viability (%), compared to the control (Table 4). 

The studies reporting the insect life cycle in the toxicity analyses provide knowledge about the total effect of a particular substance [48]. The larval viability is a parameter directly proportional to caterpillar mortality, thus these results can be correlated (Figure 9).

Several factors can affect insect survival and development, such as temperature, humidity, photoperiod, food, and exposure to toxic agents. These factors can promote sublethal effects, including *the* non-hatching of eggs, morphological structural anomalies, non-emergence of adults, and fecundity reduction. Silver nanoparticles can affect insect physiology, reducing fertility and survival, according to previously observed in the *Drosophila sp.* (Diptera: *Drosophilidae*) [1,49,50]. The results presented a modification of both the life cycle and larval survival of *P. xylostella* submitted to silver nanoparticles. The chronic effect was not observed after the change from the larval to the pupal phase.

## 4. Conclusions

In this study, a sustainable green synthesis approach of silver nanoparticles mediated by entomopathogenic fungi was described to be applicable to insect control. This work highlighted the potential action of entomopathogenic fungi extracts in the reduction and stabilization of silver nanoparticles. The insecticidal efficacy of silver nanoparticles synthesized in the presence of *I. fumosorosea* against *P. xylostella* was also demonstrated. Silver nanoparticles presented toxicity against *P. xylostella caterpillars*, showing close values compared to a commercial chemical pesticide; it may thus be considered an advantageous alternative in the control of *P. xylostella* populations.

## Figures and Tables

**Table 1:** Entomopathogenic fungi isolates used in this study.

Isolate	Specie	Origin	Collection Site/Host
1260	*Beauveria bassiana*	Laboratory of Pathology of Insects (USP), Piracicaba, (SP-Brazil)	Insect *Leptopharsa larvae*
SE109		Laboratory of Biotechnological Pest Control (Sergipetec), São Cristovão (SE-Brazil)	Soil
SE202	*Metarhizium anisopliae*	Laboratory of Biotechnological Pest Control (Sergipetec), São Cristovão (SE-Brazil)	Soil
SE301	*Isaria fumosorosea*	Laboratory of Biotechnological Pest Control (Sergipetec), São Cristovão (SE-Brazil)	Soil

**Table 2:** Effect of silver nitrate (AgNO_3_) concentration on the average diameter and polydispersity of silver nanoparticles synthesized with aqueous extracts of entomopathogenic fungi.

Isolate	AgNO_3_	Diameter (nm) *	PI *^•^
1260	1 mM	202.60 ± 20.25	0.54 ± 0.08
	3 mM	224.83 ± 61.87	0.52 ± 0.04
	5 mM	257.07 ± 17.86	0.55 ± 0.06
SE109	1 mM	172.77 ± 59.73	0.54 ± 0.07
	3 mM	121.37 ± 20.68	0.50 ± 0.05
	5 mM	119.48 ± 6.11	0.47 ± 0.04
SE202	1 mM	103.97 ± 6.30	0.27 ± 0.05
	3 mM	122.44 ± 4.34	0.41 ± 0.03
	5 mM	135.32 ± 23.39	0.54 ± 0.07
SE301	1 mM	129.03 ± 30.51	0.55 ± 0.09
	3 mM	86.26 ± 8.19	0.37 ± 0.05
	5 mM	119.39 ± 26.69	0.54 ± 0.06

* Mean values with standard error. ^•^PI = Polydispersity index.

**Table 3:** Estimation of lethal concentration (LC) of silver nanoparticles synthesized with the fungus *Isaria fumosorosea* on *Plutella xylostella* (AgNPs) and Deltamethrin.

Treatment	*N*	LC_30_	LC_50_	LC_90_	Slope	χ^2^	P
AgNPs	741	0.144 (0.104–0.182)	0.691 (0.627–0.762)	2.011 (1.684–2.550)	2.762 (±0.231)	7.634	0.054
Deltamethrin	750	0.009 (0.005–0,013)	0.301 (0.238–0.391)	3.427 (2.211–6.033)	1.214 (±0.088)	4.222	0.238

Lethal concentration values in mg/mL, followed by lower and upper limit (*p* < 0.05); Slope value, followed by standard error; *N*: Total insects used in the analysis; χ^2^: Chi-square test; P: Significance of χ^2^.

**Table 4:** Analysis of the impact of silver nanoparticles synthesized with the fungus *Isaria fumosorosea* (LC_50_) on the development of *Plutella xylostella*.

AgNPs Concentration (mg/mL)	Life Cycle Phase			
	Larvae		Pupae	
	Duration (Days)	Viability (%)	Duration (Days)	Viability (%)
0.0	10.8 ± 1.44	91.14 ± 1.76	8.6 ± 1.34	79.56 ± 7.06
0.7	10.8 ± 1.04	21.74 ± 11.04	7.2 ± 1.09	77.95 ± 14.90
T	0.000	13.879	1.807	0.219
*p*	1	0.000	0.108	0.832

Average values of duration and viability, followed by standard error; Analysis of variance (ANOVA) for comparing values in the column (*p* < 0.05); T = Student test; *p* = Calculated significance.

## Data Availability

Not applicable.
